# Prospective cohort study of influenza vaccine effectiveness among healthcare personnel in Lima, Peru: Estudio Vacuna de Influenza Peru, 2016‐2018

**DOI:** 10.1111/irv.12737

**Published:** 2020-04-05

**Authors:** Meredith G. Wesley, Giselle Soto, Carmen Sofia Arriola, Miriam Gonzales, Gabriella Newes‐Adeyi, Candice Romero, Vic Veguilla, Min Z. Levine, Maria Silva, Jill M. Ferdinands, Fatimah S. Dawood, Sue B. Reynolds, Avital Hirsch, Mark Katz, Eduardo Matos, Eduardo Ticona, Juan Castro, Maria Castillo, Eduar Bravo, Angela Cheung, Rachel Phadnis, Emily Toth Martin, Yeny Tinoco, Joan Manuel Neyra Quijandria, Eduardo Azziz‐Baumgartner, Mark G. Thompson, the VIP Cohort Study Working Group, Suryaprakash Sambhara, Shivaprakash Gangappa, Ryan E. Malosh, Christopher Flygare, Weiping Cao, Margarita Mishina, Young Moo Yoo, Christopher N. Mores, Wesley R. Campbell

**Affiliations:** ^1^ Influenza Division Centers for Disease Control and Prevention Atlanta GA USA; ^2^ U.S. Naval Medical Research Unit No. 6 Bellavista Peru; ^3^ Abt Associates Atlanta GA USA; ^4^ Chief Physician's Office Clalit Health Services Clalit Research Institute Tel Aviv Israel; ^5^ Hospital Nacional Arzobispo Loayza Lima Peru; ^6^ Hospital Nacional Dos de Mayo Lima Peru; ^7^ Hospital Nacional Daniel Alcides Carrion Lima Peru; ^8^ Instituto Nacional de Salud del Niño Lima Peru; ^9^ Medical School Universidad Peruana Cayetano Heredia Lima Peru; ^10^ University of Michigan School of Public Health Ann Arbor MI USA

**Keywords:** healthcare personnel, influenza, influenza vaccine

## Abstract

**Background:**

The Estudio Vacuna de Influenza Peru (VIP) cohort aims to describe the frequency of influenza virus infection, identify predictors of vaccine acceptance, examine the effects of repeated influenza vaccination on immunogenicity, and evaluate influenza vaccine effectiveness among HCP.

**Methods:**

The VIP cohort prospectively followed HCP in Lima, Peru, during the 2016‐2018 influenza seasons; a fourth year is ongoing. Participants contribute blood samples before and after the influenza season and after influenza vaccination (for vaccinees). Weekly surveillance is conducted to identify acute respiratory or febrile illnesses (ARFI). When an ARFI is identified, participants self‐collect nasal swabs that are tested for influenza viruses by real‐time reverse transcriptase‐polymerase chain reaction. Influenza vaccination status and 5‐year vaccination history are ascertained. We analyzed recruitment and enrollment results for 2016‐2018 and surveillance participation for 2016‐2017.

**Results:**

In the first 3 years of the cohort, VIP successfully contacted 92% of potential participants, enrolled 76% of eligible HCP, and retained >90% of participants across years. About half of participants are medical assistants (54%), and most provide “hands‐on” medical care (76%). Sixty‐nine percent and 52% of participants completed surveillance for >70% of weeks in years 1 and 2, respectively. Fewer weeks of completed surveillance was associated with older age (≥50 years), being a medical assistant, self‐rated health of fair or poor, and not receiving the influenza vaccine during the current season (*P*‐values < .05).

**Conclusions:**

The VIP cohort provides an opportunity to address knowledge gaps about influenza virus infection, vaccination uptake, effectiveness and immunogenicity among HCP.

## 1 INTRODUCTION

A multi‐year, prospective cohort study of healthcare personnel (HCP) in Lima, Peru, is underway, named Estudio Vacuna de Influenza Peru (VIP). Here, we summarize the objectives and design, results of recruitment during the first 3 years of the study, and rates of participation in active surveillance during the first 2 years of the study.

A meta‐analysis of studies of seasonal influenza estimated that 1/5 HCP are infected with influenza virus annually, based on serologic and clinical testing.^1^ Estimates of influenza virus infection among HCP vary widely depending on the extent of active surveillance and whether studies relied on serologic^2^ or molecular diagnostics.^3^, ^4^ Healthcare personnel are believed to be at increased risk because of frequent patient contact. They may also transmit influenza to their patients, though the extent of these risks is unclear.^5^ Because HCP often work while ill,^3^, ^6^, ^7^ more information is needed on the number and types of contacts HCP may have with patients while HCP are symptomatic with influenza and other viral infections.^3^, ^6^, ^7^, ^8^ Recent research suggests that certain subgroups of HCP, such as those that perform aerosol‐generating procedures, may be at heightened risk of infection with respiratory pathogens including influenza.^9^ Our cohort study was designed to address gaps in our knowledge of influenza burden and impact among HCP. The first objective of the VIP Cohort is to describe the frequency of influenza virus infections among HCP, including acute illnesses and asymptomatic infections.

Vaccination of HCP against influenza virus infection is an important component of infection control in healthcare settings,^10^ but relatively low uptake among HCP outside the United States remains a topic of international concern and debate.^11^, ^12^, ^13^ Although numerous studies of the knowledge, attitudes, and practices (KAP) associated with influenza vaccine acceptance have been conducted among HCP in high‐income countries,^14^, ^15^, ^16^, ^17^ less is known about barriers to vaccine acceptance among HCP in low‐ and middle‐income countries.^13^, ^15^, ^18^ The second objective is to identify predictors of vaccine acceptance and hesitancy in HCP.

Studies of influenza vaccine immunogenicity among HCP have demonstrated that repeated vaccination can blunt the antibody response to hemagglutinin^19^, ^20^ and neuraminidase.^21^ Further research is needed to examine how influenza vaccination across multiple seasons may affect immunogenicity^22^ and how these effects are mediated by specific humoral^20^ and cell‐mediated immune responses.^22^ The third objective is to examine how repeated influenza vaccination may modify immunogenicity.

Although recent reviews confirm that seasonal influenza vaccine is moderately effective in reducing the risk of illness among adults,^23^ there are limited data regarding the value of vaccine for HCP. To date, the only randomized controlled trial of influenza vaccine efficacy among HCPs relied on serologic outcomes,^24^ which are biased among vaccinees and may inflate influenza vaccine effectiveness (IVE) estimates.^25^, ^26^ Reports of reduced IVE among frequent vaccinees in some studies^20^, ^22^, ^27^ and seasons^28^ make it important to examine IVE among HCP, a population that receives frequent annual influenza vaccinations in the United States. Few data are available about the value of influenza vaccine in reducing missed work due to infection or reducing frequency of time worked while ill.^5^, ^8^ Given that influenza vaccine may only reduce the risk of influenza illness by 40%‐60% during years with a good match between circulating and vaccine viruses, further research is needed on whether factors like age, patient‐care responsibilities, and the use of personal protective equipment (PPE) modify the risk of vaccine failure. Limited research suggests that vaccination may also modify illness duration and severity among those who develop influenza illness despite vaccination.^8^, ^29^, ^30^, ^31^ The fourth objective of the VIP Cohort is to evaluate IVE in preventing influenza illness and associated missed work and working while ill. See Appendix S1 for more detail on study objectives.

## 2 METHODS

### 2.1 Setting

The VIP Cohort recruited HCP in Lima, Peru, at Dos de Mayo National Hospital, Cayetano Heredia National Hospital, and Daniel Alcides Carrión National Hospital in 2016 and expanded to include National Institute of Child Health (Del Niño) and Archbishop Loayza Hospital in 2017 (Table S1).

### 2.2 Eligibility criteria

Eligible participants are HCP aged ≥18 years, working ≥30 hours/week, with routine, direct patient contact and must have been employed by the hospital for ≥1 year. Similar to previous definitions for HCP,^32^ we include a variety of HCP, including direct care providers, allied‐health workers, and non‐clinical personnel. Participants are ineligible if they received the current seasonal influenza vaccine prior to enrollment.

### 2.3 Recruitment strategy

To minimize potential selection biases, HCP are invited to join the cohort using a stratified sampling strategy. We categorize potential participants at each hospital into 18 strata by sex, three age groups, and three occupational categories. To ensure the cohort includes participants with all combinations of sex, age, and occupation, we set a goal of ≥50 participants in each strata. We set goals for total recruitment in year 1 of 1200, year 2 of 2800, and year 3 of 2400, and set minimum enrollment goals per study hospital (Appendix S1).

### 2.4 Enrollment

Participants complete an enrollment survey when they enter the cohort and complete follow‐up surveys at the end of season and start of season for their remaining time in the cohort. The enrollment survey gathers information on sociodemographic characteristics, work responsibilities, health status, health behaviors, and KAP regarding influenza illness and vaccination (Appendix S1). Influenza vaccination history for five prior years is documented by self‐report at enrollment and extracted from each hospital's employee vaccination registry (Appendix S1, Table S2).

### 2.5 Active surveillance

Based on previous surveillance for laboratory‐confirmed influenza virus infection in Lima,^33^ we conduct active surveillance for ARFI during ~20 weeks each year. The start of active surveillance is informed by historical trends and early reports of laboratory‐confirmed influenza virus infection from clinical and public health sources in Lima.^33^

During the influenza season, participants receive twice‐weekly short‐message‐service (SMS) text messages to confirm whether they had an acute illness with one or more of the following symptoms within the past 7 days: cough, runny nose, body aches, or feverishness. Upon illness identification, staff conduct an acute illness survey and participants contribute a self‐collected nasal swab. Staff conduct a follow‐up survey at illness resolution. To verify surveillance completeness and mitigate information bias, the end‐of‐season survey asks participants whether any illness was missed during the season (Appendix S1, Figure S1).

### 2.6 Influenza virus infection detection

The primary study outcome is ARFI associated with influenza virus infection confirmed by rRT‐PCR. Specimens are tested by NAMRU‐6 Laboratory for influenza A and B viruses, subtypes and lineages using rRT‐PCR assays, with standard protocols, primers, probes, and reagents supplied by US CDC's International Reagent Resource (IRR) (Appendix S1).

### 2.7 Blood specimens

All participants contribute 10 mL of whole blood at enrollment and 5 mL at the start of session and end of season; vaccinees also provide 5 mL approximately 28 days (21‐42 days) after vaccination. A subset of participants provide an additional 10 mL of whole blood at start of season and end of season and approximately 7 days post‐vaccination (for vaccinees) for extraction of peripheral blood mononuclear cells (PBMCs). See Appendix S1 and Figure S2 for more information on laboratory testing.

### 2.8 Data management

Data collection and management were conducted using REDCap (Research Electronic Data Capture), a browser‐based metadata‐driven software system^34^ (Appendix S1).

### 2.9 Statistical power

We expect 1500‐2000 HCP participants to enroll each year with approximately 50% enrolling in multiple years. Thus, we assumed we would observe at least 5000 person‐seasons, approximately 30% HCP vaccination coverage and 7% influenza illness attack rate, with *α* = 0.05, we are 80% powered to estimate a true VE of approximately 30% and to estimate a difference in cumulative incidence between vaccinated and unvaccinated HCP of approximately 2.3 cases per 100 HCP. A higher VE and/or greater difference in cumulative incidence by vaccination status would increase the statistical power. Models, such as a generalized estimating equation, that take into account repeated observations should improve statistical power. See Appendix S1 for detail on statistical analysis plans.

### 2.10 Statistical analysis to date

To assess the stratified recruitment approach, we evaluated the proportion of HCP who fully enrolled out of all eligible HCP. Full enrollment is defined as providing informed consent, completing the enrollment survey and contributing the enrollment blood sample. We compared full enrollment stratified by major recruitment categories in the 18 recruitment strata (sex by occupation by age) using chi‐square tests and used multivariable logistic regression to model full study enrollment as a function of these five factors.

To describe performance of surveillance activities in years 1 and 2, we examined the proportion of participants who completed surveillance participation each week, defined as completion of surveillance questions. Participants known to have an ongoing illness and therefore ineligible for contact during a week were counted among completed surveillance events for that week. We used multivariable linear regression to predict the percentage of all surveillance weeks with completed contact as a function of the major recruitment variables (sex, age at enrollment categories, occupational categories, and hospital). Surveillance data from year 1 and year 2 were evaluated separately. Variables with fewer than 10 missing responses are denoted on the tables; data were not imputed for these analyses.

### 2.11 Ethical approval and ethical considerations

The study protocol and procedures were reviewed and approved by seven institutional review boards including NAMRU‐6, each study hospital and by Abt Associates (coordinating institution for US CDC). All participants completed written informed consent. Small gifts were given to participants at study milestones. Given the research nature of the laboratory methods and time delays in batch testing, rRT‐PCR results were not available to participants and did not inform decisions regarding their medical care or approval to return‐to‐work.

## 3 FINDINGS

### 3.1 Recruitment and retention

**Figure 1:**
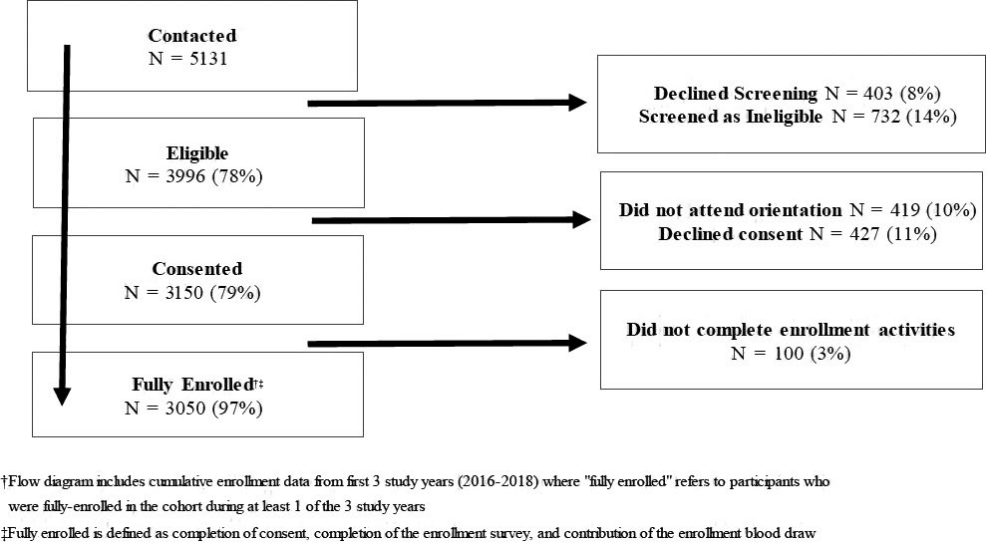
Flow diagram of participant recruitment and enrollment, VIP cohort, 2016‐2018

The recruitment flow diagram for years 1‐3 is presented in Figure 1. We successfully contacted 92% (4728/5131) of potential participants (Table S3). Of eligible HCP, 76% (3050/3996) consented and enrolled (Table 1). We met our recruitment goal of enrolling ≥50 HCP in 17 of the 18 recruitment strata. There were statistically significant differences between eligible HCP who enrolled versus refused by year, sex, age, occupation, and hospital. With the exception of occupation, these factors continued to be associated with the odds of enrollment in a multivariable model. Agreement to enroll increased with each study year, was higher among females and those aged <50 years, and varied between hospitals (range = 57%‐93%).

**Table 1:** Predictors of Healthcare Personnel Enrollment by Demographic and Occupational Strata during Recruitment, VIP Cohort, 2016‐2018

		Enrollment of eligible HCP			Predictors of full enrollment among eligible HCP^a^	
		Fully enrolled^a^	Eligible	Row%	aOR^b^	95%CI
*Major Recruitment Categories*						
Cumulative		3050	/3996	76		
Year						
2016		1145	/1895	60	Ref.	
2017		1795	/1989	90	5.7*	4.4‐7.5
2018		110	/112	98	49.6*	11.7‐210.5
Sex						
Male		864	/1173	74	Ref.	
Female		2186	/2823	77	1.3*	1.1‐1.5
Age						
18‐34		952	/1170	81	1.6*	1.3‐2.0
35‐49		1231	/1588	78	1.5*	1.2‐1.8
≥50		867	/1238	70	Ref.	
Occupation						
Physicians		433	/628	69	Ref.	
Nurses/technicians		983	/1322	74	0.9	0.7‐1.2
Assistants		1634	/2046	80	1.1	0.9‐1.4
Hospitals						
Dos de Mayo		744	/1112	67	1.9*	1.5‐2.3
Cayetano Heredia		756	/961	79	2.5*	2.0‐3.2
Carrión		326	/576	57	Ref.	
Del Niño		596	/638	93	2.9*	1.9‐4.3
Loayza		628	/709	89	1.2	0.9‐1.8
*Recruitment strata across hospitals and years*						
Sex and age	Occupation					
Males						
18‐34	Physicians	74	/99	75		
18‐34	Nurses/technicians	54	/70	77		
18‐34	Assistants	158	/194	81		
35‐49	Physicians	107	/159	67		
35‐49	Nurses/technicians	63	/85	74		
35‐49	Assistants	196	/223	88		
≥50	Physicians	65	/131	50		
≥50	Nurses/technicians	25	/39	64		
≥50	Assistants	122	/173	71		
Females						
18‐34	Physicians	65	/74	88		
18‐34	Nurses/technicians	266	/327	81		
18‐34	Assistants	335	/406	83		
35‐49	Physicians	71	/90	79		
35‐49	Nurses/technicians	346	/471	73		
35‐49	Assistants	448	/560	80		
≥50	Physicians	51	/75	68		
≥50	Nurses/technicians	229	/330	69		
≥50	Assistants	375	/490	77		

Abbreviations: 95% Confidence interval; aOR, Adjusted odds ratio CI.

^a^ Fully enrolled defined as informed consent, completion of enrollment survey, and contribution of enrollment blood sample.

^b^ Logistic regression model of full study enrollment as a function of year, sex, age at enrollment, occupation, and hospital.

* *P*‐value < .05.

Information on study retention is currently available through the start of year 3 (Table S5). Of year 1 enrollees, 90% (1035/1145) completed study activities and continued participation in year 2; of year 2 enrollees, 94% (2672/2831) continued into year 3. The most common reasons for study withdrawal were discontinuation of employment at the study hospital (43%, 115/269) or unwillingness to contribute a blood sample (36%, 96/269). Although study withdrawal is low across sociodemographic groups (Table S5), statistically significant differences were noted by hospital (range = 6%‐17%), and withdrawal is statistically higher among younger participants, physicians, and those who reported never receiving an influenza vaccine.

### 3.2 Characteristics of enrolled participants

Characteristics of the 3050 HCP enrolled during years 1‐3 are in Table 2 (by year in Table S4). Most cohort participants were female (72%) and aged <50 years old (72%). Approximately half were medical assistants (54%), while 32% were nurses and technologists and 14% were physicians. Most report providing “hands‐on” care (76%) and regularly performing aerosol‐generating procedures (58%). Although most participants were healthy, 21% reports ≥1 chronic medical condition, and 20% describe their overall health as only “fair” or “poor.” Most (85%) report having received the influenza vaccine at least once before enrollment.

**Table 2:** Characteristics of fully enrolled participants, VIP cohort, 2016‐2018 (N = 3050)

	Total
	N = 3050^a^
	n (Col.%)
Hospital	
Dos de Mayo	744 (24)
Cayetano Heredia	756 (25)
Carrión	326 (11)
Del Niño	596 (20)
Loayza	628 (21)
Sex	
Male	864 (28)
Female	2186 (72)
Age	
18‐34	952 (31)
35‐49	1231 (40)
≥50	867 (28)
By occupation	
Physicians	433 (14)
Nurses/technicians	983 (32)
Assistants	1634 (54)
Marital status	
Married or cohabitating	1644 (54)
Never married, separated, divorced or widowed	1406 (46)
Household monthly income (Soles)	
≤3000 S	1534 (50)
3001‐6000 S	617 (20)
>6001 S	451 (15)
Refused	448 (15)
Others in household^b^, median (IQR)	3 (2, 4)
Self‐rated overall health^b^	
Excellent	138 (5)
Very good	637 (21)
Good	1678 (55)
Fair/poor	595 (20)
Current chronic medical condition^c^	
Yes	633 (21)
No	2417 (79)
Ever received influenza vaccine^d^	
Yes	2559 (84)
No	410 (13)
Don't know^e^	81 (3)
Days in pain interfere with activities, median (IQR)^b^, ^f^	0 (0, 2)
Days healthy and full of energy, median (IQR)^b^, ^f^	26 (20, 30)
Hands‐on clinician	
Yes	2329 (76)
No	721 (24)
Conducts aerosol producing procedures^g^	
Yes	1762 (58)
No	1288 (42)
Number of years seeing patients, median (IQR)^b^	12 (5, 22)

Abbreviation: IQR, Interquartile range.

^a^ Fully enrolled defined as informed consent, completion of enrollment survey, and contribution of enrollment blood sample.

^b^ <10 missing responses.

^c^ Currently receiving medical care for ≥1 of asthma, cancer, lung condition, diabetes, heart condition, high blood pressure, immunosuppression/problem with immune system, kidney disease, neurologic problem, and other.

^d^ Self‐reported vaccination history.

^e^ "Don't know" (n = 21), missing (n = 60).

^f^ Possible responses range from 0‐30 d.

^g^ Regularly administers ≥1 of the following: collects respiratory swab, collects sputum specimen, administers medication using nebulizer, applies nasal cannula, applies oxygen facemask, performs tracheal intubation, inserts nasogastric tube, performs manual ventilation, performs suction of fluids, performs chest physiotherapy, and performs bedside bronchoscopy.

### 3.3 Surveillance participation

**Figure 2:**
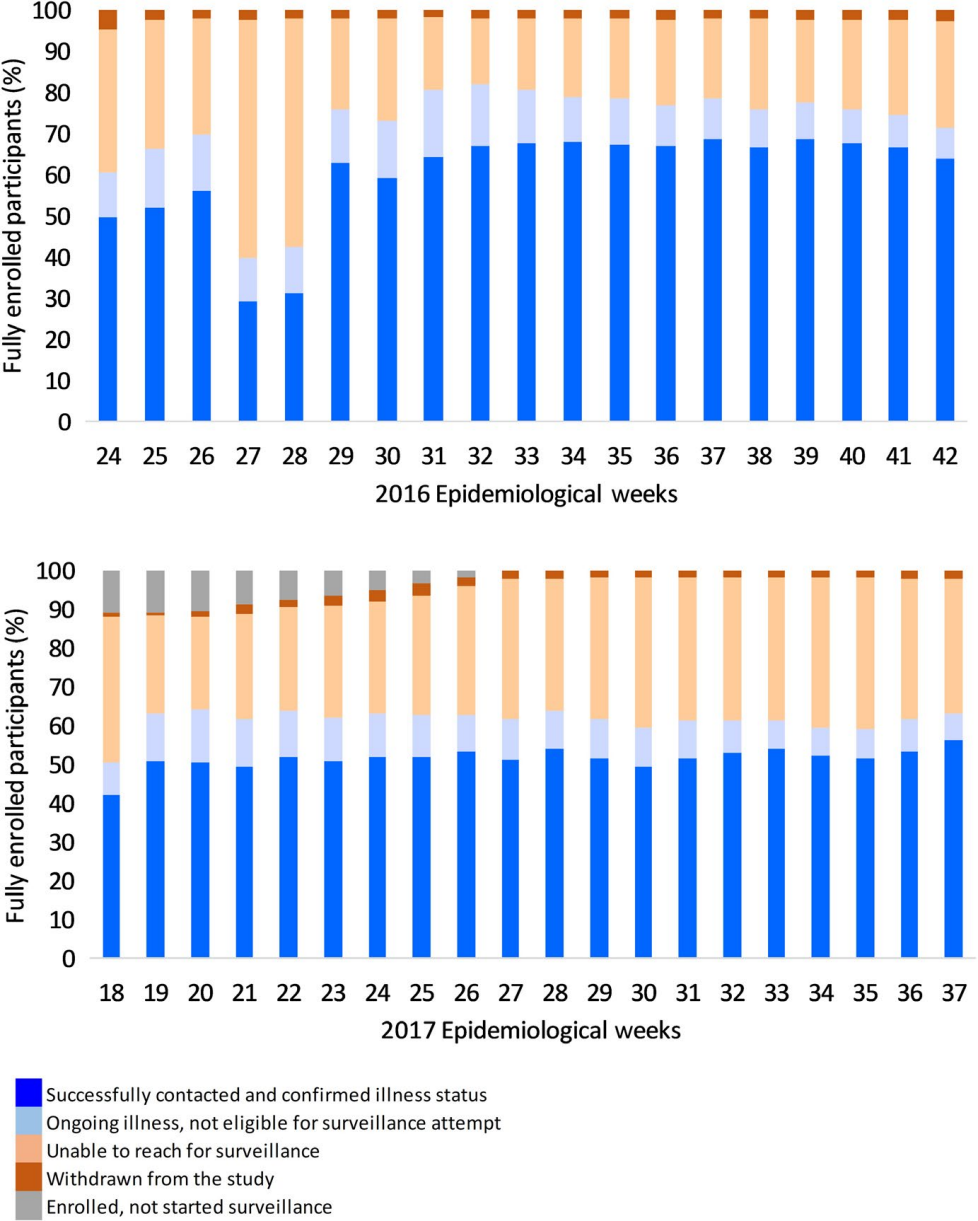
Participation in active surveillance by epidemiological week, VIP cohort, 2016 (top), 2017 (bottom)

Results on active surveillance participation are available for the 19 weeks of surveillance in year 1 (epi‐weeks 23‐41, 2016) and 20 weeks in year 2 (epi‐weeks 18‐ 37, 2017). Figure 2 presents the percentage of participants in four categories by week: (a) successfully confirmed illness status; (b) ongoing illness, thus excluded from routine contacts; (c) unable to contact for surveillance; and (d) withdrawn. Categories 1‐2 combined represent “completed surveillance.” Technical problems with the SMS systems led to relatively low contacts for 2 weeks in year 1 (weeks 27 and 28). In year 2, surveillance completion was relatively low in the first week because a substantial number of participants had enrolled but had not started surveillance. With the exception of these weeks, surveillance was completed by >60% of participants for all weeks in years 1 and 2 (range = 61%‐82%).

**Figure 3:**
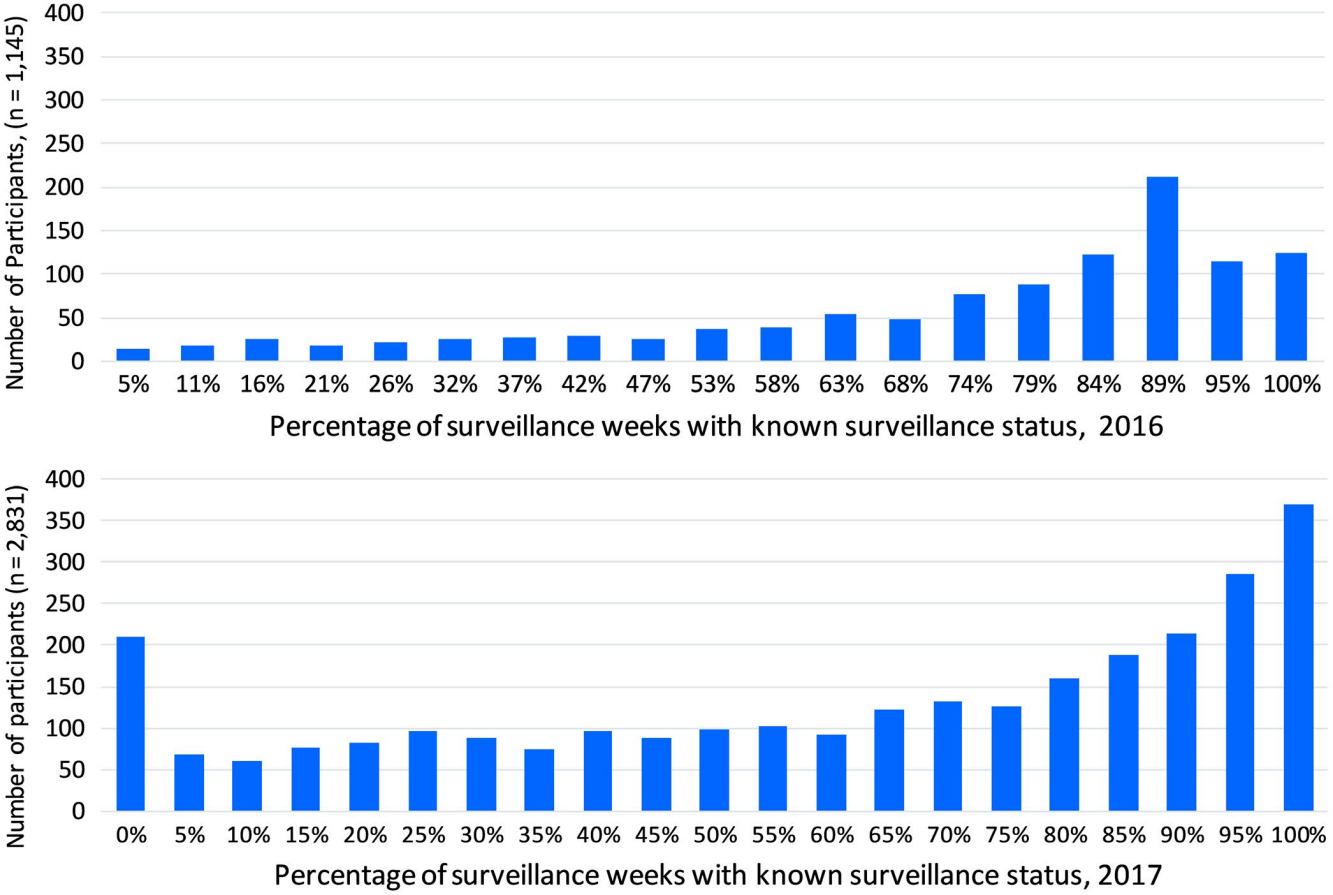
Participation in active surveillance by proportion of successful surveillance weeks, VIP cohort, 2016 (top), 2017 (bottom)

At the participant level, the mean percentage of weeks with completed surveillance was statistically higher in year 1 (71.6%) than year 2 (61.5%) (*F*‐ratio[1] = 84.79, *P* < .001), though there was variability in surveillance completion across weeks in both years (Figure 3). A small percentage of participants failed to complete any weekly surveillance reports: 2% (25/1145) in year 1 and 7% (210/2831) in year 2. Over half of participants completed surveillance for >70% of weeks: 69% (786/1145) in year 1 and 52% (1475/2871) in year 2. For each year, we examined the percentage of surveillance weeks completed as a function of hospital, sex, age, occupation, self‐rated health, chronic medical condition, and influenza vaccination during the season, using multivariable linear regression (Table 3). In both years, adjusting for all variables simultaneously, completed surveillance weeks was statistically higher for participants aged 35‐49 years, those in “very good” self‐rated health and those who received the influenza vaccine, and was statistically lower for medical assistants and at some study hospitals. Completed surveillance was also higher among females but this was only statistically significant in year 2.

**Table 3:** Factors associated with successful surveillance participation (% of total weeks) using multivariable linear regression, VIP cohort, 2016‐2017

	2016 N = 1145		2017 N = 2831	
	Estimate	95%CI	Estimate	95%CI
Intercept	81.00	(74.52, 87.90)	53.02	(46.7, 59.31)
Hospital				
Dos de Mayo	−11.65*	(−15.70, −7.60)	1.83*	(−2.78, 6.45)
Cayetano Heredia	−10.37*	(−14.66, −6.08)	4.80	(0.22, 9.38)
Carrión	Ref.		Ref.	
Del Niño	N/A		2.18	(−2.37, 6.74)
Loayza	N/A		−10.26*	(−14.99, −5.53)
Sex				
Male	Ref.		Ref.	
Female	3.46	(−0.04, 6.96)	4.40*	(1.50, 7.30)
Age				
18‐34	3.41	(−0.54, 7.35)	0.06*	(0.02, 0.09)
35‐49	4.55*	(1.01, 8.10)	10.60*	(7.53, 13.67)
≥50	Ref.		Ref.	
By Occupation				
Physicians	Ref.		Ref.	
Nurses/technicians	−0.05*	(−0.09, 0.00)	−0.01	(−0.05, 0.03)
Assistants	−0.15*	(−0.19, −0.11)	−0.13*	(−0.17, −0.09)
Self‐rated overall health^a^				
Excellent	1.98	(−5.62, 9.59)	1.52	(−5.07, 8.11)
Very good	5.09*	(0.32, 9.86)	9.96*	(5.93, 14.00)
Good	2.40	(−1.15, 6.83)	6.12*	(2.91, 9.45)
Fair/Poor	Ref.		Ref.	
Current chronic medical condition^b^				
Yes	−1.16	(−4.72, 2.41)	0.97	(−2.19, 4.14)
No	Ref.		Ref.	
Vaccination during study year				
Yes	3.37*	(0.38, 6.36)	4.41*	(1.66, 7.17)
No	Ref.		Ref.	

Abbreviations: CI, 95% Confidence interval; N/A, Not study site in year 1; β, Unstandardized regression coefficient.

^a^ <10 missing responses.

^b^ Currently receiving medical care for ≥1 of asthma, cancer, lung condition, diabetes, heart condition, high blood pressure, immunosuppression/problem with immune system, kidney disease, neurologic problem, and other.

* *P* < .05.

In the end‐of‐season survey, a small percentage of participants reported that they had failed to report at least one possible ARFI as part of surveillance: 10% (112/1145) in year 1 and 7% (205/2831) in year 2. Participants who said they forgot to report an illness had fewer weeks of completed surveillance in year 1 versus those who did not forget (Mean[SD] = 65.6%[27.7%] vs 74.1%[24.67%], *F*‐ratio = 11.6[1], *P* < .001) and year 2 (58.6%[33.0%] vs 63.2%[32.6%]; 3.8[1] *P* = .052).

## 4 DISCUSSION

The VIP Cohort is poised to address knowledge gaps regarding the burden of laboratory‐confirmed influenza illness and the preventive value of influenza vaccines among HCP. This study is unique in its ability to assess the risk of rRT‐PCR‐confirmed influenza illness and immune response to infection and influenza vaccination among HCP who received Southern‐hemisphere influenza vaccines for several seasons. The study includes serology on all participants which affords the opportunity to quantify sub‐clinical or asymptomatic infections that may not be captured by PCR‐based testing. Insights provided by such results may be particularly timely given recent efforts by the World Health Organization to enhance influenza vaccine coverage among HCP, especially in middle‐income countries, to protect HCP and their patients during seasonal influenza epidemics and increase pandemic preparedness.^35^

A strength of this study is the ability to describe all stages of recruitment starting with a known source population denominator. Because we can quantify the source population, we can assess potential selection bias, which is an important source of potential bias in observational IVE studies.^36^, ^37^ The VIP Cohort study successfully reached 92% of potential participants, enrolled 76% of eligible HCP, and has retained ≥90% of participants between years. This represents very high overall participation rates compared to earlier studies of HCP^17^, ^38^ and other cohort studies of adults.^39^, ^40^ Statistically significant differences in enrollment between hospitals and by sex, age, and occupation are consistent with differences noted in a previous HCP cohort in the United States^17^ and highlight the importance of the study's stratified recruitment strategy to insure participants with combinations of these characteristics are represented. The target enrollment of ≥50 HCP per 18 recruitment strata was met for all strata except for the least common combination, male nurses aged ≥50. The stratified recruitment strategy generated variability in participant characteristics that can aid in adjusted IVE models, assessment of possible IVE effect modification, and estimating the weighted incidence of influenza virus infection in the source population of HCP across hospitals.

During the first 2 years, over half of the participants completed ≥70% of surveillance weeks. This is higher than surveillance participation reported in similar studies of acute respiratory illness,^40^ but reports of participation at this level of detail are rarely published. Despite use of SMS text messaging and other modes of communication for surveillance, illness status was uncertain in about 30% of participants per week, on average. In years 1 and 2, 10% and 7% of participants, respectively, reported that they failed to report an acute illness during the season. Gaps in surveillance data create potential for information bias; in a multivariable model, we found male sex, age ≥50, occupation as a nurse/technician or medical assistant, self‐rated overall health as “fair” or “poor,” and having not received the vaccination in the current season were associated with missing more weeks of surveillance. Nonetheless, the ability to quantify this missing information and address it in statistical models for IVE and influenza virus infection incidence represent a strength of the study.

This study has several other limitations. Like all studies of IVE and influenza incidence, the ability to broadly generalize results is limited by the unpredictability of circulating virus types and potential for mismatch between vaccine components and circulating strains in any year. Although conducting the study in Peru allows us to examine IVE in a middle‐income and Southern‐hemisphere country, where data on IVE are limited, the generalizability of findings to the United States and other countries is unknown. Additionally, the overall intensity and impact of influenza seasons are variable, and low influenza activity in a study season could negatively affect our ability to precisely estimate IVE and incidence. There is potential for bias in recall of information collected by self‐report, including vaccination history and details about illness severity and duration.

This study provides a unique opportunity to characterize and understand influenza illness among HCP and the impact of influenza illness on work in healthcare settings. In this context, we can better understand the role influenza vaccines play in protecting HCP from becoming infected, missing work, or working while sick, and the serologic response produced by influenza vaccines in a repeatedly vaccinated population.

## AUTHOR CONTRIBUTIONS

**Meredith Wesley: **Data curation (equal); Formal analysis (lead); Writing – original draft (lead); Writing – review & editing (equal). **Giselle Soto: **Conceptualization (equal); Data curation (equal); Investigation (lead); Methodology (equal); Writing – review & editing (equal). **Carmen Arriola: **Conceptualization (equal); Investigation (equal); Methodology (equal); Writing – review & editing (equal). **Miriam Gonzales: **Data curation (equal); Investigation (equal); Project administration (equal); Writing – review & editing (equal). **Gabriella Newes‐Adeyi: **Conceptualization (equal); Investigation (equal); Methodology (equal); Project administration (equal); Supervision (equal); Writing – review & editing (equal). **Candice Romero: **Data curation (equal); Investigation (equal); Project administration (equal); Writing – review & editing (equal). **Vic Veguilla: **Investigation (equal); Methodology (equal); Writing – review & editing (equal). **Min Levine: **Investigation (equal); Methodology (equal); Writing – review & editing (equal). **Maria Silva: **Investigation (equal); Methodology (equal); Writing – review & editing (supporting). **Jill Ferdinands: **Conceptualization (equal); Investigation (equal); Methodology (equal); Writing – review & editing (equal). **Fatimah Dawood: **Conceptualization (equal); Investigation (equal); Methodology (equal); Writing – review & editing (equal). **Sue Reynolds: **Formal analysis (equal); Methodology (equal); Writing – review & editing (equal). **Avital Hirsch: **Methodology (equal); Writing – review & editing (equal). **Mark Katz: **Methodology (equal); Writing – review & editing (equal). **Eduardo Matos: **Investigation (equal); Project administration (equal); Writing – review & editing (equal). **Eduardo Ticona: **Investigation (equal); Project administration (equal); Writing – review & editing (equal). **Juan Castro: **Investigation (equal); Project administration (equal); Writing – review & editing (equal). **Maria Castillo: **Investigation (equal); Project administration (equal); Writing – review & editing (equal). **Eduar Bravo: **Investigation (equal); Project administration (equal); Writing – review & editing (equal). **Angela Cheung: **Data curation (equal); Writing – review & editing (equal). **Rachael Phadnis: **Data curation (equal); Writing – review & editing (equal). **Emily Martin: **Investigation (equal); Methodology (equal); Writing – review & editing (equal). **Yeny Tinoco: **Investigation (equal); Methodology (equal); Project administration (equal); Writing – review & editing (equal). **Joan Manuel Neyra Quijandria: **Data curation (equal); Methodology (equal); Project administration (equal); Writing – review & editing (equal). **Eduardo Azziz‐Baumgartner: **Conceptualization (equal); Investigation (equal); Methodology (equal); Writing – review & editing (equal). **Mark Thompson: **Conceptualization (lead); Investigation (lead); Methodology (lead); Supervision (lead); Writing – original draft (equal); Writing‐review & editing (lead).

## DISCLAIMERS

The views expressed in this manuscript reflect the results of research conducted by the authors and do not necessarily reflect the official policy or position of the Centers for Disease Control and Prevention, the Department of the Navy, Department of Defense, nor the US Government.

Some authors are employee of the US Government. This work was prepared as part of their official duties. Title 17, USC, §105 provides that copyright protection under this title is not available for any work of the US Government. Title 17, USC, §101 defines a US Government work as a work prepared by a military Service member or employee of the US Government as part of that person's official duties.

## Supporting information

Fig S1

Fig S2

Supplementary Material

## APPENDIX 1

VIP Cohort Study Working Group: Suryaprakash Sambhara (Influenza Division, Centers for Disease Control and Prevention, Atlanta, GA, USA), Shivaprakash Gangappa, Ryan E. Malosh (University of Michigan School of Public Health, Ann Arbor, MI, USA), Christopher Flygare (Abt Associates, Atlanta, GA, USA), Weiping Cao (Influenza Division, Centers for Disease Control and Prevention, Atlanta, GA, USA), Margarita Mishina (Influenza Division, Centers for Disease Control and Prevention, Atlanta, GA, USA), Young Moo Yoo (Battelle, Atlanta, GA, USA), Christopher N. Mores (Milken Institute School of Public Health, The George Washington University, Washington, DC, USA), Wesley R. Campbell (Division of Infectious Diseases, Department of Internal Medicine, Walter Reed National Military Medical Center, Bethesda, MD, USA).
