# Supplementary material for: Prospective cohort study of influenza vaccine effectiveness among healthcare personnel in Lima, Peru: Estudio Vacuna de Influenza Peru, 2016‐2018
Source: Influenza Other Respir Viruses. 2020 Apr 5;14(4):391–402. doi: 10.1111/irv.12737 (PMC7298283; doi:10.1111/irv.12737)
Supplement: Supplementary file 3 — Supplementary Material [file IRV-14-391-s003.docx]

Prospective cohort study of influenza vaccine effectiveness among healthcare personnel in Lima, Peru: Estudio Vacuna de Influenza Peru (VIP), 2016-2018

APPENDIX S1

Supplemental Methods

1. Study Objectives….………………………………………………………………………………..2
2. Recruitment Methods………….………………………………………………………….…..……3
3. Knowledge, Attitudes and Practices Measure………………………………..………………….…4
4. Influenza Vaccination Status Ascertainment……………………………...……………………….6
5. Surveillance Methods………………………………………………………………………………6
6. Influenza Virus Infection Detection……………………………..…………………………………7
7. Blood Specimen Collection.………………………………………………………………...…..…8
8. Laboratory Testing…………………………………………….……………………………….…..8
9. Data Management…………….………………………………………………………………..…10
10. Statistical Considerations………………………………..……………………………………..…10
11. STROBE Statement………………………………………………………………………………13
12. References………………………………………………………………………………………...16
13. Supplemental Table S1……………………………………………………………………………17
14. Supplemental Table S2…………………………………………………………………….………18
15. Supplemental Table S3……………………………………………………….……………………19
16. Supplemental Table S4………………………………………………………………………….…20
17. Supplemental Table S5………………………………………………………….…………………22

**1. Study Objectives**

The VIP Cohort study has 4 main objectives. Elements of the study design support specific study objectives as listed below.

1. Describe the frequency of influenza virus infections among HCP, including acute illnesses and asymptomatic infections.

- Conduct active surveillance using the following broad case definition for acute respiratory or febrile illness (ARFI): “illness with cough, runny nose, body aches, or feverishness in the past 7 days”;
- Use both molecular and serologic diagnostics to identify clinical and subclinical influenza virus infections;
- Compare the frequency of influenza illness by sex, age, underlying health status, occupation, patient populations served, hours providing direct patient care, and performance of aerosol-generating procedures;
- Examine the association between influenza illness and self-reported adherence to hand washing and personal protective equipment (PPE) guidelines;

1. Identify predictors of vaccine acceptance and hesitancy.

- Describe how KAP vary by demographic characteristics, with the frequency of influenza vaccination during the 5 years prior to enrollment and predict subsequent vaccination during participation in the Peru cohort;
- Examine how knowledge and concern about potential transmission of influenza to patients is associated with influenza vaccination and other preventive behaviors, including hand washing and use of PPE.

1. Examine how repeated influenza vaccination may modify immunogenicity.

- Examine the extent to which repeated influenza vaccination is associated with titers of antibody to HA as quantified by hemagglutination inhibition (HAI);
  - Vaccination history is considered for 5 years prior to the study and for 2-3 years of cohort participation;
- Examine whether any link between repeated vaccination and HAI titers varies by participants’ sex, age, birth cohort, or underlying health status, or by participants’ repertoire of antibody responses to viruses in prior vaccines and circulating strains;
- In a subset of participants who provide peripheral blood mononuclear cells (PBMCs) before and after vaccination, examine whether repeated prior vaccination is associated with suppression of B cell and T cell immunogenicity.

1. Evaluate IVE in preventing influenza illness and associated missed work and working while ill.

- Estimate IVE in preventing real time reverse transcriptase polymerase chain reaction (rRT-PCR)-confirmed influenza illness among HCP;
- Estimate IVE in reducing hours of missed work due to influenza illness and hours working while ill with influenza;
- Examine whether IVE of current season vaccination is modified by frequent vs. infrequent prior vaccinations;
- Among HCP with influenza illness, examine whether symptom severity and illness duration are lower among vaccinated vs. unvaccinated HCP.

**2. Recruitment Methods**

Stratified recruitment was employed for several reasons. First, having a mixture of participant characteristics (age, sex, and occupation) and hospital settings, allows us to examine the extent to which these factors may be associated with influenza vaccination propensity and influenza incidence. To the extent that any of these factors are potential confounders, having a mixture of participant characteristics may allow us to examine and adjust for these factors in VE estimates. Second, a stratified recruitment model facilitates explicit monitoring of and assessing for selection bias. Third, a stratified sample facilitates weighting incidence estimates from the observed cohort in a systematic way in order to create an incidence estimate for the source population of all HCP in the study hospitals.

Strata were created by sex, age group, and occupation group, given prior research that indicated these factors were associated with differences in the likelihood of participating in a prospective cohort study, receiving influenza vaccines, differences in immune responses to influenza vaccines, and differential exposure to influenza viruses in medical settings and at home.

Recruitment was conducted in three waves. During the first wave at each hospital, we identified a minimum number of HCP to recruit per recruitment strata. For strata with less common combinations of characteristics (e.g., male nurses age ≥50 years), this meant we attempted to contact all HCP with those characteristics. For strata with a large number of HCP (e.g., female nurses aged 18-34 years), a random subset was selected. We used a combination of personal contact at the hospital, telephone calls, and emails in our attempt to reach >80% of the potential participants identified. During the second wave, we made additional attempts to contact HCP who had not been reached during the first wave, especially for strata with limited numbers of HCP; for more populous strata, we randomly selected additional HCP to contact. Finally, in wave three, we accepted volunteers if necessary to meet the total sample goals for each hospital and cumulative goals per year.

**3. Knowledge, Attitudes and Practices Measures**

Participants were randomized to receive one of two versions of the KAP items, each that emphasize different constructs, and a subset of KAP items from all constructs are asked to every participant. Participants were asked to rate their intention to be vaccinated in the future (using a standardized 7-point verbal description of likelihood)^1^ as part of every study survey. KAP constructs were measured with multiple items that examine different aspects of the conceptual content, drawn from previously validated items^2-5^. Different response scales (e.g., 5 point agree to disagree scale vs. other Likert-like scales) were used to optimize measured variability and minimize measurement error (e.g., errors due to response sets such as consistently picking the highest or lowest option). The constructs measured in each set of items is described below:

- Core Items
  - Readiness
  - Safety
  - Accurate knowledge about influenza virus transmission to patients
  - Perceived risks and susceptibility to disease
  - Perceived severity of disease
  - Shot/needle concerns
  - Prior experience [with influenza vaccination]
  - Convenience and access to the vaccine
  - Affective attitudes and emotional expectations
  - Perceived benefits and effectiveness of action
  - Perceived social norms
  - Social influence of co-workers/family/friends
- Random Version 1
  - Readiness
  - Safety
  - Accurate knowledge about influenza virus transmission to patients
  - Perceived risks and susceptibility to disease
  - Perceived severity of disease
  - Shot/needle concerns
  - Brand or source concerns
  - Prior experience [with influenza vaccination]
- Random Version 2
  - Convenience and access to the vaccine
  - Affective attitudes and emotional expectations
  - Perceived benefits and effectiveness of action
  - Perceived social norms
  - Social influence of co-workers/family/friends

**4. Influenza Vaccination Status Ascertainment**

At study enrollment, participants were asked about their history of influenza vaccination during the prior 5 years including how many of the 5 years they received the vaccine and in which of the 5 prior seasons they received it and any associated side effects.

Study staff accessed hospital vaccination records, as available, to verify self-reported vaccination history going back up to 5 previous years and receipt of vaccine during the study seasons. They recorded vaccination date, vaccine type, manufacturer, and lot numbers if available. Those participants who chose to receive influenza vaccine outside of the site institution were asked to provide a copy of their vaccination record to study staff, if available.

**5. Active Surveillance**

During the influenza season, participants received twice-weekly short message service (SMS) text messages asking them to confirm whether or not they have had an acute illness with one or more of the following symptoms within the past 7 days: cough, runny nose, body aches, or feverishness. Study staff called participants by telephone if they fail to reply to two consecutive text messages, and participants were asked to contact staff directly when they experience symptoms of acute illness. Once an illness was identified by any of these methods, staff follow the methods below. See Supplemental Figure B for additional detail.

- Staff conducted an acute illness survey by telephone that confirmed the illness meets the case definition, and verified the date of illness onset (Supplemental Figure A).
  - The acute illness survey assessed the occurrence of 17 symptoms and the perceived severity of each symptom/the overall illness (mild, moderate, or severe).
- Participants were asked to self-collect a nasal swab using a self-swabbing kit that included illustrated instructions, a nasal mid-turbinate flocked swab, and a cryovial with room temperature commercial transport medium.
- Staff arranged to pick up the swab kit during the same day or the following morning.
- Seven days after illness onset, staff telephoned to ask if the participant is “no longer sick;” if the illness was not resolved, inquiries were repeated every 48 hours until the illness was resolved or up to 3 contacts (on days 9, 11, and 13 post illness onset).
- At illness resolution, staff completed an illness follow-up survey by telephone, which assessed any new symptoms that have occurred since the initial survey and whether medical care was provided during the illness.
- For each day a participant reported having symptoms, starting with the date of illness onset (as reported during the acute illness survey) through the final day of illness (as reported at the illness follow-up survey), participants were asked: (a) if they worked at the hospital, and if so the number of hours they worked; (b) if they missed any scheduled work, and if so the hours missed; (c) to rate their ability to do normal activities and responsibilities at work (0-100%); (d) to rate their ability to do normal activities and responsibilities outside of work (0-100%).
- To verify surveillance completeness and mitigate information bias, the end-of-season survey asks participants: “We understand that we probably missed some illnesses because participants were too busy or simply forgot to report them. During the weeks when we sent SMS texts to you twice a week, did you have an illness (with feverishness, cough, runny nose, or body aches) that you forgot to report or tell the staff about?”

**6. Influenza Virus Detection**

Respiratory specimens were temporarily stored in cooler boxes at 4-8^0^C and delivered by staff every afternoon during weekdays to the Virology Laboratory of the Naval Medical Research Unit No. 6 (NAMRU-6) facility in Lima, where they were aliquoted (into 1 working aliquot and 3 stored aliquots) and then frozen in a -80^0^C freezer. After one freeze-thaw cycle, specimens are tested by NAMRU-6 Laboratory for influenza A and B viruses and influenza A subtypes and B lineages using real time reverse transcriptase polymerase chain reaction (rRT-PCR) assays, with protocols, primers, probes, and reagents supplied by US CDC’s International Reagent Resource (IRR). Influenza B lineages are identified using CDC’s genotyping protocol and reagents from IRR.

**7. Blood Specimen Collection**

Starting in year 3, we collected 10mL at all blood draws. Sera were extracted and distributed to ~4 aliquots and frozen in a -20^0^C freezer at a second study laboratory (Centro de Investigaciones Tecnológicas, Biomédicas y Medio Ambientales [CITBM]).

A subset of participants were asked to provide an additional 10mL of heparinized whole blood at enrollment, approximately 7 days post-vaccination (for vaccinees), and at the end of season for isolation of peripheral blood mononuclear cells (PBMCs). At the CITBM laboratory, PBMC samples were centrifuged, underwent cell count, were diluted 1-5x10^6^ cells/ ml and frozen at -80^o^C, and then placed in liquid nitrogen within 24 hours. PBMC samples were used for assays focused on cell-mediated immunity (CMI) at a US CDC-designated laboratory.

**8. Laboratory Testing**

*Hemagglutination Inhibition Assay (HAI)*

The Hemagglutination Inhibition (HAI) Assay was used to detect the presence of neutralizing influenza antibodies in serum. HAI to IIV components and to influenza circulating strains were performed by a US CDC-designated laboratory using standard methods^6^ as described previously^7, 8^. Egg-grown wild type viruses were supplied by US CDC’s IRR. Preparation of serum samples included (a) treatment with receptor-destroying enzyme to remove nonspecific inhibitors, and (b) removal of nonspecific agglutinins by serum adsorption with packed red blood cells (RBCs). Standard 0.5% turkey RBCs were prepared for A(H1N1)pdm09 antigens and ether-treated B influenza antigens. Given indications that neuraminidase (NA) of new antigenic clusters of A(H3N2) (since 2014) have acquired the ability to bind to RBCs, modified HAI assays were conducted for A(H3N2) antigens using Guinea Pig red blood cells in the presence of the antiviral oseltamivir carboxylate, which inhibits influenza NA.

Serum was diluted 2-fold starting from 1:10. The HAI titer was the reciprocal of the serum dilution in the last well with complete HI. The geometric mean titer (GMT) from duplicate results was reported; HAI <10 was considered 5 for GMT calculation.

Supplemental Figure B presents the southern hemisphere, trivalent influenza vaccine components from 2011-2018 and the selected target antigens for HAI testing in this study.

*Cell-mediated immunity (CMI) assessments*

PBMC will be used for assessment of cell-mediated immune responses to vaccination. Briefly, multi-parametric flow cytometry will be performed to measure the frequency of T cells post-vaccination by examining surface markers of activation and secretion of interferon-gamma (IFN-gamma), interleukin 2 (IL-2), and Tumor Necrosis Factor-alpha (TNF-alpha) by intra cellular cytokine staining (ICCS), antibody secreting plasmablasts and memory B cells to hemagglutinin (HA), to vaccine strains at baseline, approximately 7 days post-vaccination and memory B cells at the end of the season^9^. In addition, B cell repertoire from plasmablasts and HA-specific clonotypes in sera collected 28 days post-vaccination will be carried out in a subset of subjects**^10, 11^.**

*Other Laboratory Tests*

Additional serologic tests were performed including multi-virus molecular diagnostic panels such as fast track diagnostic (FTD) testing and film array testing, neurominidase inhibition assay (NAI) and microneutralization assay (MN). Vaccine products from study years 2 and 3 were tested by Isotope Dilution Mass Spectrometry to quantify and compare antigenicity.

*Isotope Dilution Mass Spectrometry (IDMS)*

IDMS was performed on a sample of vaccine products used in study hospitals during years 2 and 3 to better understand the quantity of antigen in the vaccine products relative to one another. The products include Vaxigrip trivalent (2017) and FluQuadri quadrivalent (2018) from Sanofi Pasteur and GC FLU trivalent (2018) from Green Cross Corporation.

**9. Data Management**

Data collection and site-level management was conducted using REDCap (Research Electronic Data Capture), a browser-based metadata-driven software system (Vanderbilt University, Nashville, TN)^12^. Most study instruments, including the recruitment log, online surveys, and laboratory results allowed for real-time data entry directly into REDCap. Surveys were designed to be self-administered electronically via the internet using home computers, personal mobile telephones, or computer tablets provided at the workplace. For illness assessments conducted by telephone, staff entered data directly into the REDCap database. Routine quality assurance monitoring was conducted locally by the project manager and centrally by the data coordinator (Abt Associates). SMS text messaging was administered by TextIt, (TextIt, Kigali, Rwanda), which uses custom decision trees to trigger exchanges with participants. Study staff performed bi-weekly exports of TextIt data and imported the data into a Microsoft Excel macro-enabled workbook to track participant responses and illness events over time.

**10. Statistical Considerations**

*Statistical analysis approach*

Standard descriptive statistics and both bivariate (unadjusted) and multivariate logistic regression (adjusted) analyses will be conducted to assess the first and second objectives. For the third objective, antibody response to vaccination will be assessed using a linear mixed effects model, with previously published methods^20^. For the fourth objective, IVE against rRT-PCR confirmed influenza illness will be assessed using adjusted multivariate log-binomial regression in a generalized estimating equation (GEE) framework. The same approach will be used to assess IVE against number of work hours missed due to influenza illness and hours working while ill with influenza (Supplemental Methods). To examine whether symptom severity and illness duration are lower among vaccinated versus unvaccinated personnel (among HCP with influenza illness), adjusted logistic regression models will be implemented. Causal mediation analysis and/or effect modification methods will be used to examine whether IVE of current season vaccination is modified by frequent versus infrequent prior vaccination.

Two main objectives of this study are to estimate the cumulative incidence and evaluate influenza vaccine effectiveness in preventing influenza illness among HCP (Supplemental Methods). To date, 3,050 HCP are fully enrolled in the study over the initial 3 years.

Since the study spanned three years, many HCP at each site participated during more than one influenza season. To account for this within-subject correlation across seasons, a generalized estimating equation (GEE) approach will be used to assess the association between PCR-confirmed influenza illness and current season influenza vaccination.

Unlike a mixed-effects model which captures correlation between observations by incorporating subject-level random effects into the model, the GEE approach treats the subject-level correlation as a nuisance parameter. Instead, GEE analysis, the subject-level correlation is incorporated directly into the variance-covariance matrix and not included as an effect in the model. Thus, GEEs provide “population-averaged” estimates of the parameters^13, 14^.

GEE uses a quasi-likelihood function to estimate the parameters of a generalized linear model when responses are correlated. Using this analytic approach, the model is specified through (1) a link function that relates the mean response to a linear predictor, (2) an assumed distribution for the response, and (3) a working correlation matrix which describes how subject-level responses are correlated. Parameter and standard error estimates from the GEE are consistent even when the covariance structure is misspecified, under mild regularity conditions. To attain valid estimates, the number of clusters (or subjects) should be somewhat large (e.g., >50)^13, 14^.

For this study, log-binomial regression within a GEE framework will be used to model the dichotomous outcome variable, PCR-confirmed influenza status. An exchangeable correlation matrix structure will be used to capture subject-level correlation. This matrix structure assumes that correlation within individuals is time invariant. Relative risk (RR) will be estimated from this model, and VE will be calculated as 1-RR. The linear predictor will consist of a combination of continuous and discrete variables. The exposure of interest is vaccination status at time of symptom onset. Any persons with unknown vaccination status will be excluded from the study.

If any statistical method is found to be unsuitable during analysis due to unexpected recruitment or seasonal effects (e.g., inadequate sample size, late availability of influenza vaccine, low participation in surveillance), alternate methods will be used. Any changes in methodology will be documented.

For estimates of influenza illness incidence, we plan to calculate incidence using the enrolled cohort as the denominator and then extrapolate the incidence to the source population of participating hospitals. Because the cohort was recruited using a random stratified sampling, we identified the number of HCP in each strata as part of recruitment. We will calculate the incidence for each strata and then use survey sampling weights to estimate the incidence for the study hospitals^15^.

**10. STROBE Statement**

|  | Item No | Recommendation | Page  No |
| --- | --- | --- | --- |
| **Title and abstract** | 1 | (*a*) Indicate the study’s design with a commonly used term in the title or the abstract | 1 |
|  |  | (*b*) Provide in the abstract an informative and balanced summary of what was done and what was found | 3 |
| Introduction | | | |
| Background/rationale | 2 | Explain the scientific background and rationale for the investigation being reported | 4-5 |
| Objectives | 3 | State specific objectives, including any pre-specified hypotheses | 4-5 |
| Methods | | | |
| Study design | 4 | Present key elements of study design early in the paper | 5-6 |
| Setting | 5 | Describe the setting, locations, and relevant dates, including periods of recruitment, exposure, follow-up, and data collection | 5 |
| Participants | 6 | (*a*) *Cohort study*—Give the eligibility criteria, and the sources and methods of selection of participants. Describe methods of follow-up  *Case-control study*—Give the eligibility criteria, and the sources and methods of case ascertainment and control selection. Give the rationale for the choice of cases and controls  *Cross-sectional study*—Give the eligibility criteria, and the sources and methods of selection of participants | 6 |
|  |  | (*b*) *Cohort study*—For matched studies, give matching criteria and number of exposed and unexposed  *Case-control study*—For matched studies, give matching criteria and the number of controls per case |  |
| Variables | 7 | Clearly define all outcomes, exposures, predictors, potential confounders, and effect modifiers. Give diagnostic criteria, if applicable | 6-8 |
| Data sources/ measurement | 8* | For each variable of interest, give sources of data and details of methods of assessment (measurement). Describe comparability of assessment methods if there is more than one group | 6-8 |
| Bias | 9 | Describe any efforts to address potential sources of bias | 8 |
| Study size | 10 | Explain how the study size was arrived at | 7 |
| Quantitative variables | 11 | Explain how quantitative variables were handled in the analyses. If applicable, describe which groupings were chosen and why | 6-8 |
| Statistical methods | 12 | (*a*) Describe all statistical methods, including those used to control for confounding | 6-8 |
|  |  | (*b*) Describe any methods used to examine subgroups and interactions | 6-8 |
|  |  | (*c*) Explain how missing data were addressed | 8 |
|  |  | (*d*) *Cohort study*—If applicable, explain how loss to follow-up was addressed  *Case-control study*—If applicable, explain how matching of cases and controls was addressed  *Cross-sectional study*—If applicable, describe analytical methods taking account of sampling strategy | 6-8 |
|  |  | (*e*) Describe any sensitivity analyses | 6-8 |

| Results | | | |
| --- | --- | --- | --- |
| Participants | 13* | (a) Report numbers of individuals at each stage of study—eg numbers potentially eligible, examined for eligibility, confirmed eligible, included in the study, completing follow-up, and analysed | 9, Table 1, Figure 1 |
|  |  | (b) Give reasons for non-participation at each stage | 9, Table 1, Figure 1 |
|  |  | (c) Consider use of a flow diagram | Figure 1 |
| Descriptive data | 14* | (a) Give characteristics of study participants (eg demographic, clinical, social) and information on exposures and potential confounders | Table 2 |
|  |  | (b) Indicate number of participants with missing data for each variable of interest | Table 2 |
|  |  | (c) *Cohort study*—Summarise follow-up time (eg, average and total amount) | 9-10 |
| Outcome data | 15* | *Cohort study*—Report numbers of outcome events or summary measures over time | 9-10 |
|  |  | *Case-control study—*Report numbers in each exposure category, or summary measures of exposure |  |
|  |  | *Cross-sectional study—*Report numbers of outcome events or summary measures |  |
| Main results | 16 | (*a*) Give unadjusted estimates and, if applicable, confounder-adjusted estimates and their precision (eg, 95% confidence interval). Make clear which confounders were adjusted for and why they were included | 9-10, Tables 1-3, Figures 2-3 |
|  |  | (*b*) Report category boundaries when continuous variables were categorized | 9-10 |
|  |  | (*c*) If relevant, consider translating estimates of relative risk into absolute risk for a meaningful time period |  |
| Other analyses | 17 | Report other analyses done—eg analyses of subgroups and interactions, and sensitivity analyses | 9-10, Tables 1-3, Figures 2-3 |
| Discussion | | | |
| Key results | 18 | Summarise key results with reference to study objectives | 11-12 |
| Limitations | 19 | Discuss limitations of the study, taking into account sources of potential bias or imprecision. Discuss both direction and magnitude of any potential bias | 11-12 |
| Interpretation | 20 | Give a cautious overall interpretation of results considering objectives, limitations, multiplicity of analyses, results from similar studies, and other relevant evidence | 11-12 |
| Generalisability | 21 | Discuss the generalisability (external validity) of the study results | 11-12 |
| Other information | | | |
| Funding | 22 | Give the source of funding and the role of the funders for the present study and, if applicable, for the original study on which the present article is based | 13 |

**References**

1. Thompson MG, Gaglani MJ, Naleway A, et al. The expected emotional benefits of influenza vaccination strongly affect pre-season intentions and subsequent vaccination among healthcare personnel. Vaccine 2012; 30:3557-65.
2. Abu-Gharbieh E, Fahmy S, Rasool BA, Khan S. Influenza vaccination: healthcare workers attitude in three Middle East countries. International journal of medical sciences 2010; 7:319-25.
3. Hollmeyer H, Hayden F, Mounts A, Buchholz U. Review: interventions to increase influenza vaccination among healthcare workers in hospitals. Influenza and other respiratory viruses 2013; 7:604-21.
4. Hollmeyer HG, Hayden F, Poland G, Buchholz U. Influenza vaccination of health care workers in hospitals--a review of studies on attitudes and predictors. Vaccine 2009; 27:3935-44.
5. Naleway AL, Henkle EM, Ball S, et al. Barriers and facilitators to influenza vaccination and vaccine coverage in a cohort of health care personnel. Am J Infect Control 2014; 42:371-5.
6. Network WHOGIS. Serological diagnosis of influenza by haemagglutination inhibition testing. In: Manual for the laboratory diagnosis and virological surveillance of influenza. Geneva, Switzerland: WHO, 2011; 59-62.
7. Pearson ML, Bridges CB, Harper SA. Influenza vaccination of health-care personnel: recommendations of the Healthcare Infection Control Practices Advisory Committee (HICPAC) and the Advisory Committee on Immunization Practices (ACIP). MMWR Recomm Rep 2006; 55:1-16.
8. Henkle E, Irving SA, Naleway AL, et al. Comparison of laboratory-confirmed influenza and noninfluenza acute respiratory illness in healthcare personnel during the 2010-2011 influenza season. Infect Control Hosp Epidemiol 2014; 35:538-46.
9. Amoah, S., Mishina, M., Praphasiri, P., Cao, W., Kim, J. H., Liepkalns, J. S., ... & Garg, S. (2019). Standard-Dose Intradermal Influenza Vaccine Elicits Cellular Immune Responses Similar to Those of Intramuscular Vaccine in Men With and Those Without HIV Infection. The Journal of Infectious Diseases.
10. Wang, B., DeKosky, B. J., Timm, M. R., Lee, J., Normandin, E., Misasi, J., ... & Niezold, T. (2018). Functional interrogation and mining of natively paired human V H: V L antibody repertoires. Nature biotechnology, 36(2), 152.
11. Lee, J., Paparoditis, P., Horton, A. P., Frühwirth, A., McDaniel, J. R., Jung, J., ... & Ippolito, G. C. (2019). Persistent antibody clonotypes dominate the serum response to influenza over multiple years and repeated vaccinations. Cell host & microbe, 25(3), 367-376.
12. Harris PA, Taylor R, Thielke R, Payne J, Gonzalez N, Conde JG. Research electronic data capture (REDCap)--a metadata-driven methodology and workflow process for providing translational research informatics support. J Biomed Inform 2009; 42:377-81.
13. Liang, K-Y., Zeger, S.L.. Longitudinal data analysis using generalized linear models. Biometrika 1986, 73(1), 13-22.
14. Hardin, J., Hilbe J., Generalized Estimating Equations. 2003. Boca Raton, FL: Chapman and Hall/CRC.
15. Lee, E. S., R. N. Forthofer, and R. J. Lorimor. 1989. Analyzing Complex Survey Data. Sage University Paper Series on Quantitative Applications in the Social Sciences, series no. 07-071. Beverly Hills, CA, and London: Sage Publications.

**10. Supplemental Tables**

| Table S1. Description of Study Hospitals and their Healthcare Personnel, VIP Cohort, 2016-2018 | | | | | | |
| --- | --- | --- | --- | --- | --- | --- |
|  |  |  |  | Approximate Number of HCP | | |
| Hospital | Type of Hospital | Population Served | Hospital Beds | Physicians | Nurses, Therapists, Technicians | Medical- Assistants |
| Dos de Mayo National Hospital | Public Tertiary Care | 4861380 | 641 | 661 | 562 | 1467 |
| Cayetano Heredia National Hospital | Public Tertiary Care | 2597553 | 394 | 475 | 1260 | 1205 |
| Daniel Alcides Carrion National Hospital | Public Tertiary Care | 1024439 | 547 | 276 | 352 | 982 |
| Natinoal Institute of Child Health | Public, Tertiary Care | 6179930 | 401 | 600 | 733 | 1531 |
| Archbishop Loayza Hospital | Public Tertiary Care | 9111000 | 802 | 477 | 681 | 1730 |
|  |  |  |  |  |  |  |

| Table S2. Key Study Variables by Domain and Information Sources, VIP Cohort, 2016-2018 | | | | |
| --- | --- | --- | --- | --- |
| Domain | **Self-reported** | | | Employee Records |
|  | Enrollment | Mid-season | End of Season |  |
| **Socio-demographic Characteristics** |  |  |  |  |
| Sex | ✓ |  |  | ✓ |
| DOB | ✓ |  |  |  |
| Number of household members | ✓ |  |  |  |
| Marital status | ✓ |  |  |  |
| Occupation | ✓ |  |  |  |
| Work responsibilities | ✓ |  |  | ✓ |
| Perception of illness | ✓ |  | ✓ |  |
| Perception of vaccines | ✓ |  | ✓ |  |
| Perceptions of missing work | ✓ |  |  |  |
| Job satisfaction | ✓ |  | ✓ |  |
|  |  |  |  |  |
| **Current Health Status, Risk Behaviors, Medications** |  |  |  |  |
| Current health | ✓ |  | ✓ |  |
| Height | ✓ |  |  |  |
| Weight | ✓ |  |  |  |
| Chronic medical conditions | ✓ |  |  |  |
| Smoking status | ✓ |  |  |  |
| Smoking history | ✓ |  |  |  |
| Pregnancy | ✓ |  |  |  |
|  |  |  |  |  |
| **Influenza Vaccination Documentation** |  |  |  |  |
| Receipt of vaccination in the current season | ✓ | ✓ | ✓ | ✓ |
| Receipt of vaccination in the prior 5 seasons | ✓ | ✓ |  | ✓ |
| Vaccine administration date |  | ✓ | ✓ | ✓ |
| Vaccine type |  | ✓ |  | ✓ |
| Vaccine manufacturer and lot (if available) |  | ✓ |  | ✓ |
| Source of documentation |  | ✓ |  | ✓ |
|  |  |  |  |  |
| **Illness** |  |  |  |  |
| Acute respiratory illness |  | ✓ | ✓ |  |
| Number of days with an absence associated with illness | | ✓ |  | ✓ |

| Table S3. Screened and Enrolled Healthcare Personnel by Demographic and Occupational Strata During Recruitment, VIP Cohort, 2016-2018 | | | | | | | | | | | |
| --- | --- | --- | --- | --- | --- | --- | --- | --- | --- | --- | --- |
|  | | Screening of Potential Participants^†^ | | | | | Enrollment of Eligible HCP | | | | |
|  | | Screened | / | Contacted | Row % | p-value | Fully Enrolled^‡^ | / | Eligible | Row % | p-value |
| **Major Recruitment Categories** | |  |  |  |  |  |  |  |  |  |  |
| Cumulative Recruitment | | 4728 | / | 5131 | (92) |  | 3050 | / | 3996 | (76) |  |
|  |  |  |  |  |  |  |  |  |  |  |  |
| Year |  |  |  |  |  | <0.001 |  |  |  |  | <0.001 |
| 2016 |  | 2601 | / | 3004 | (87) |  | 1145 | / | 1895 | (60) |  |
| 2017 |  | 2015 | / | 2015 | (100) |  | 1795 | / | 1989 | (90) |  |
| 2018 |  | 112 | / | 112 | (100) |  | 110 | / | 112 | (98) |  |
|  |  |  |  |  |  |  |  |  |  |  |  |
| Sex |  |  |  |  |  | 0.871 |  |  |  |  | <0.001 |
| Male |  | 1430 | / | 1551 | (92) |  | 864 | / | 1173 | (74) |  |
| Female |  | 3298 | / | 3580 | (92) |  | 2186 | / | 2823 | (77) |  |
|  |  |  |  |  |  |  |  |  |  |  |  |
| Age |  |  |  |  |  | <0.001 |  |  |  |  | <0.001 |
| 18-34 |  | 1388 | / | 1425 | (97) |  | 952 | / | 1170 | (81) |  |
| 35-49 |  | 1838 | / | 1989 | (92) |  | 1231 | / | 1588 | (78) |  |
| ≥50 |  | 1502 | / | 1717 | (87) |  | 867 | / | 1238 | (70) |  |
|  |  |  |  |  |  |  |  |  |  |  |  |
| Occupation |  |  |  |  |  | 0.038 |  |  |  |  | <0.001 |
| Physicians |  | 805 | / | 889 | (91) |  | 433 | / | 628 | (69) |  |
| Nurses/technicians |  | 1544 | / | 1686 | (92) |  | 983 | / | 1322 | (74) |  |
| Assistants |  | 2379 | / | 2556 | (93) |  | 1634 | / | 2046 | (80) |  |
|  |  |  |  |  |  |  |  |  |  |  |  |
| Hospitals |  |  |  |  |  | <0.001 |  |  |  |  | <0.001 |
| Dos de Mayo |  | 1323 | / | 1603 | (83) |  | 744 | / | 1112 | (67) |  |
| Cayetano Heredia |  | 1193 | / | 1288 | (93) |  | 756 | / | 961 | (79) |  |
| Carrión |  | 856 | / | 884 | (97) |  | 326 | / | 576 | (57) |  |
| Del Niño |  | 640 | / | 640 | (100) |  | 596 | / | 638 | (93) |  |
| Loayza |  | 716 | / | 716 | (100) |  | 628 | / | 709 | (89) |  |
|  |  |  |  |  |  |  |  |  |  |  |  |
| Sex and Age | Occupation |  |  |  |  |  |  |  |  |  |  |
| Males |  |  |  |  |  | <0.001 |  |  |  |  | <0.001 |
| 18-34 | Physicians | 125 | / | 129 | (97) |  | 74 | / | 99 | (75) |  |
| 18-34 | Nurses/technicians | 82 | / | 83 | (99) |  | 54 | / | 70 | (77) |  |
| 18-34 | Assistants | 236 | / | 242 | (98) |  | 158 | / | 194 | (81) |  |
|  |  |  |  |  |  |  |  |  |  |  |  |
| 35-49 | Physicians | 205 | / | 229 | (90) |  | 107 | / | 159 | (67) |  |
| 35-49 | Nurses/technicians | 98 | / | 108 | (91) |  | 63 | / | 85 | (74) |  |
| 35-49 | Assistants | 26 | / | 276 | (95) |  | 196 | / | 223 | (88) |  |
|  |  |  |  |  |  |  |  |  |  |  |  |
| ≥50 | Physicians | 168 | / | 201 | (84) |  | 65 | / | 131 | (50) |  |
| ≥50 | Nurses/technicians | 46 | / | 47 | (98) |  | 25 | / | 39 | (64) |  |
| ≥50 | Assistants | 207 | / | 236 | (88) |  | 122 | / | 173 | (71) |  |
| Females |  |  |  |  |  | <0.001 |  |  |  |  | <0.001 |
| 18-34 | Physicians | 92 | / | 94 | (86) |  | 65 | / | 74 | (88) |  |
| 18-34 | Nurses/technicians | 392 | / | 400 | (98) |  | 266 | / | 327 | (81) |  |
| 18-34 | Assistants | 461 | / | 477 | (97) |  | 335 | / | 406 | (83) |  |
|  |  |  |  |  |  |  |  |  |  |  |  |
| 35-49 | Physicians | 118 | / | 129 | (91) |  | 71 | / | 90 | (79) |  |
| 35-49 | Nurses/technicians | 530 | / | 587 | (90) |  | 346 | / | 471 | (73) |  |
| 35-49 | Assistants | 624 | / | 660 | (95) |  | 448 | / | 560 | (80) |  |
|  |  |  |  |  |  |  |  |  |  |  |  |
| ≥50 | Physicians | 97 | / | 107 | (91) |  | 51 | / | 75 | (68) |  |
| ≥50 | Nurses/technicians | 396 | / | 461 | (86) |  | 229 | / | 330 | (69) |  |
| ≥50 | Assistants | 588 | / | 665 | (88) |  | 375 | / | 490 | (77) |  |
|  |  |  |  |  |  |  |  |  |  |  |  |
| ^†^ Up to five attempts were made by telephone, email, or in person visits to contact potential participants | | | | | | | | | | | |
| ^‡^ Fully enrolled is defined as completion of consent, completion of the enrollment survey and contribution of the enrollment blood draw | | | | | | | | | | | |

| Table S4. Characteristics of Participants Fully Enrolled in at least One Study Year by Year of Enrollment, VIP Cohort, 2016-2018 (N=3,050) | | | | | | | | | |
| --- | --- | --- | --- | --- | --- | --- | --- | --- | --- |
|  |  | | Year of Enrollment | | | | | |  |
|  | Total | | 2016 | | 2017 | | 2018 | | p-value^‡^ |
|  | N=3,050 | | N=1,145 | | N=1,795 | | N=110 | |  |
|  | n | Col.% | n | Col% | n | Col% | n | Col% |  |
| Hospital |  |  |  |  |  |  |  |  | <0.001 |
| Dos de Mayo | 744 | (24) | 561 | (49) | 183 | (10) | 0 | (0) |  |
| Cayetano Heredia | 756 | (25) | 373 | (33) | 383 | (21) | 0 | (0) |  |
| Carrión | 326 | (11) | 211 | (18) | 115 | (6) | 0 | (0) |  |
| Del Niño | 596 | (20) | 0 | (0) | 596 | (33) | 0 | (0) |  |
| Loayza | 628 | (21) | 0 | (0) | 518 | (29) | 110 | (100) |  |
|  |  |  |  |  |  |  |  |  |  |
| Sex |  |  |  |  |  |  |  |  | 0.240 |
| Male | 864 | (28) | 321 | (28) | 504 | (28) | 39 | (35) |  |
| Female | 2186 | (72) | 824 | (72) | 1291 | (72) | 71 | (65) |  |
|  |  |  |  |  |  |  |  |  |  |
| Age |  |  |  |  |  |  |  |  | 0.259 |
| 18-34 | 952 | (31) | 335 | (29) | 586 | (33) | 31 | (28) |  |
| 35-49 | 1231 | (40) | 464 | (41) | 720 | (40) | 47 | (43) |  |
| ≥50 | 867 | (28) | 346 | (30) | 489 | (27) | 32 | (29) |  |
|  |  |  |  |  |  |  |  |  |  |
| By Occupation |  |  |  |  |  |  |  |  | <0.001 |
| Physicians | 433 | (14) | 227 | (20) | 204 | (11) | 2 | (2) |  |
| Nurses/technicians | 983 | (32) | 401 | (35) | 557 | (31) | 25 | (23) |  |
| Assistants | 1634 | (54) | 517 | (45) | 1034 | (58) | 83 | (75) |  |
|  |  |  |  |  |  |  |  |  |  |
| Marital status |  |  |  |  |  |  |  |  | 0.144 |
| Married or  cohabitating | 1644 | (54) | 643 | (56) | 945 | (53) | 56 | (51) |  |
| Never married, separated,  divorced or widowed | 1406 | (46) | 502 | (44) | 850 | (47) | 54 | (49) |  |
|  |  |  |  |  |  |  |  |  |  |
| Household monthly income |  |  |  |  |  |  |  |  | <0.001 |
| ≤3000 S | 1534 | (50) | 532 | (46) | 941 | (52) | 61 | (55) |  |
| 3001-6000 S | 617 | (20) | 249 | (22) | 351 | (20) | 17 | (15) |  |
| >6001 S | 451 | (15) | 219 | (19) | 227 | (13) | 5 | (5) |  |
| Refused | 448 | (15) | 145 | (13) | 276 | (15) | 27 | (25) |  |
|  |  |  |  |  |  |  |  |  |  |
| Others in household^§^, median (IQR) | 3 | (2,4) | 3 | (2,4) | 3 | (2,4) | 3 | (2,5) | 0.061 |
|  |  |  |  |  |  |  |  |  |  |
| Self-rated overall health^§^ |  |  |  |  |  |  |  |  | 0.102 |
| Excellent | 138 | (5) | 54 | (5) | 81 | (5) | 3 | (3) |  |
| Very good | 637 | (21) | 268 | (23) | 347 | (19) | 22 | (20) |  |
| Good | 1678 | (55) | 623 | (54) | 994 | (55) | 61 | (55) |  |
| Fair/Poor | 595 | (20) | 200 | (17) | 371 | (21) | 24 | (22) |  |
|  |  |  |  |  |  |  |  |  |  |
| Current chronic medical  condition^\|\|^ |  |  |  |  |  |  |  |  | 0.001 |
| Yes | 633 | (21) | 277 | (24) | 339 | (19) | 17 | (15) |  |
| No | 2417 | (79) | 868 | (76) | 1456 | (81) | 93 | (85) |  |
|  |  |  |  |  |  |  |  |  |  |
| Ever received influenza vaccine^¶^ |  |  |  |  |  |  |  |  | <0.001 |
| Yes | 2559 | (84) | 984 | (86) | 1498 | (83) | 77 | (70) |  |
| No | 410 | (13) | 144 | (13) | 244 | (14) | 22 | (20) |  |
| Don't know^#^ | 81 | (3) | 17 | (1) | 53 | (3) | 11 | (10) |  |
|  |  |  |  |  |  |  |  |  |  |
| Days in pain interfere with activities, median (IQR)^§,††^ | 0 | (0,2) | 0 | (0,2) | 0 | (0,3) | 0 | (0,2) | 0.003 |
|  |  |  |  |  |  |  |  |  |  |
| Days healthy and full of energy, median (IQR)^§,††^ | 26 | (20,30) | 25 | (20,29) | 27 | (20,30) | 29 | (25,30) | <0.001 |
|  |  |  |  |  |  |  |  |  |  |
| Hands-on Clinician |  |  |  |  |  |  |  |  | <0.001 |
| Yes | 2329 | (76) | 878 | (77) | 1384 | (77) | 67 | (61) |  |
| No | 721 | (24) | 267 | (23) | 411 | (23) | 43 | (39) |  |
|  |  |  |  |  |  |  |  |  |  |
| Conducts aerosol producing procedures^‡‡^ |  |  |  |  |  |  |  |  | <0.001 |
| Yes | 1762 | (58) | 760 | (66) | 960 | (53) | 42 | (38) |  |
| No | 1288 | (42) | 385 | (34) | 835 | (47) | 68 | (62) |  |
|  |  |  |  |  |  |  |  |  |  |
| Number of years seeing patients, median (IQR)^§^ | 12 | (5,22) | 13 | (6,24) | 11 | (5,21) | 10 | (4,20) | 0.002 |
|  |  |  |  |  |  |  |  |  |  |
| IQR=Interquartile range | | | | | | | | | |
| †Fully enrolled defined as informed consent, completion of enrollment survey and contribution of enrollment blood sample | | | | | | | | | |
| ‡Wilcoxon rank sum test of significance between medians (continuous); Chi-square test of significance between frequencies (categorical) | | | | | | | | | |
| §<10 missing responses | | | | | | | | | |
| \|\|Reports currently receiving medical care for ≥1 of: asthma, cancer, lung condition, diabetes, heart condition, high blood pressure, immunosuppression/problem with immune system, kidney disease, neurologic problem, other | | | | | | | | | |
| ¶Self-reported vaccination history | | | | | | | | | |
| #"Don't know" (n=21), missing (n=60) | | | | | | | | | |
| ††Possible responses range from 0-30 days | | | | | | | | | |
| ‡‡Participant affirms regularly administering at least one of the following procedures: collect a respiratory specimen using a swab, collect a sputum specimen, administer medication using a nebulizer, apply nasal cannula, apply oxygen face mask, perform tracheal intubation, insert a nasogastric tube, perform manual ventilation, perform suction of fluids or secretions, perform chest physiotherapy, perform bedside bronchoscopy | | | | | | | | | |

| Table S5. Characteristics of Participants who Withdraw Mid-Season or Do Not Return for Subsequent Season, VIP Cohort, 2016-2017 (N=2,940) | | | | | |
| --- | --- | --- | --- | --- | --- |
|  | Fully Enrolled^†^ in either Year 1, Year 2 or Both | | | | |
|  | Complete Participation without Withdrawal | | Withdrew During Study Year or Between Years | |  |
|  | N= | 2671 | N= | 269 |  |
|  | n | Row % | n | Row % | p-value^‡^ |
| Year |  |  |  |  | 0.512 |
| 2016 | 1035 | (90) | 110 | (10) |  |
| 2017 | 1636 | (91) | 159 | (9) |  |
|  |  |  |  |  |  |
| Sex |  |  |  |  | 0.101 |
| Male | 738 | (89) | 87 | (11) |  |
| Female | 1933 | (91) | 182 | (9) |  |
|  |  |  |  |  |  |
| Age |  |  |  |  | <0.001 |
| 18-34 | 806 | (88) | 113 | (12) |  |
| 35-49 | 1087 | (92) | 98 | (8) |  |
| ≥50 | 778 | (93) | 58 | (7) |  |
|  |  |  |  |  |  |
| Occupation |  |  |  |  | 0.013 |
| Physicians | 377 | (87) | 54 | (13) |  |
| Nurses/technicians | 886 | (92) | 73 | (8) |  |
| Assistants | 1408 | (91) | 142 | (9) |  |
|  |  |  |  |  |  |
| Hospital |  |  |  |  | <0.001 |
| Dos de Mayo | 672 | (90) | 72 | (10) |  |
| Cayetano Heredia | 696 | (92) | 60 | (8) |  |
| Carrión | 271 | (83) | 55 | (17) |  |
| Del Niño | 547 | (92) | 49 | (8) |  |
| Loayza | 485 | (94) | 33 | (6) |  |
|  |  |  |  |  |  |
| Marital status |  |  |  |  | 0.581 |
| Married or cohabitating | 1447 | (91) | 141 | (9) |  |
| Never married, separated, divorced or  widowed | 1224 | (91) | 128 | (9) |  |
|  |  |  |  |  |  |
| Household monthly income |  |  |  |  | 0.408 |
| ≤3000 S | 1339 | (91) | 135 | (9) |  |
| 3001 - 6000 S | 555 | (92) | 46 | (8) |  |
| >6001 S | 397 | (89) | 47 | (11) |  |
| Refused | 380 | (90) | 41 | (10) |  |
|  |  |  |  |  |  |
| Others in household^§^, median (IQR) | 3 | (2, 4) | 3 | (2, 4) | 0.212 |
|  |  |  |  |  |  |
| Self-rated overall health^§^ |  |  |  |  | 0.372 |
| Excellent | 516 | (90) | 55 | (10) |  |
| Very good | 1480 | (92) | 137 | (8) |  |
| Good | 556 | (90) | 59 | (10) |  |
| Fair/Poor | 118 | (87) | 17 | (13) |  |
|  |  |  |  |  |  |
| Current chronic medical condition^\|\|^ |  |  |  |  | 0.242 |
| Yes | 568 | (92) | 49 | (8) |  |
| No | 2103 | (91) | 220 | (9) |  |
|  |  |  |  |  |  |
| Ever received influenza vaccine^¶^ |  |  |  |  | 0.007 |
| Yes | 2272 | (92) | 210 | (8) |  |
| No | 341 | (88) | 47 | (12) |  |
| Don't know^#^ | 13 | (76) | 4 | (24) |  |
|  |  |  |  |  |  |
| Days in pain interfere with activities, median (IQR) ^§,††^ | 0 | (0, 2) | 0 | (0, 2) | 0.459 |
|  |  |  |  |  |  |
| Days healthy and full of energy, median (IQR) ^§,††^ | 26 | (20, 30) | 25 | (20, 30) | 0.145 |
|  |  |  |  |  |  |
| Hands-on Clinician |  |  |  |  | <0.001 |
| Yes | 2175 | (92) | 183 | (8) |  |
| No | 496 | (85) | 86 | (15) |  |
|  |  |  |  |  |  |
| Conducts aerosol producing procedures^‡‡^ |  |  |  |  | 0.007 |
| Yes | 1576 | (92) | 136 | (8) |  |
| No | 1095 | (89) | 133 | (11) |  |
|  |  |  |  |  |  |
| Number of years seeing patients, median (IQR) ^§^ | 12 | (6, 22) | 7 | (3, 17) | <0.001 |
|  |  |  |  |  |  |
| IQR=Interquartile range | | | | | |
| †Fully enrolled defined as informed consent, completion of enrollment survey and contribution of enrollment blood sample | | | | | |
| ‡Wilcoxon rank sum test of significance between medians (continuous); Chi-square test of significance between frequencies (categorical) | | | | | |
| §<10 missing responses | | | | | |
| \|\|Reports currently receiving medical care for ≥1 of: asthma, cancer, lung condition, diabetes, heart condition, high blood pressure, immunosuppression/problem with immune system, kidney disease, neurologic problem, other | | | | | |
| ¶Self-reported vaccination history | | | | | |
| #"Don't know" (n=21), missing (n=60) | | | | | |
| ††Possible responses range from 0-30 days | | | | | |
| ‡‡Participant affirms regularly administering at least one of the following procedures: collect a respiratory specimen using a swab, collect a sputum specimen, administer medication using a nebulizer, apply nasal cannula, apply oxygen face mask, perform tracheal intubation, insert a nasogastric tube, perform manual ventilation, perform suction of fluids or secretions, perform chest physiotherapy, perform bedside bronchoscopy | | | | | |
|  | | | | | |
|  | | | | | |
|  | | | | | |
