# Supplementary material for: Prospective cohort study of influenza vaccine effectiveness among healthcare personnel in Lima, Peru: Estudio Vacuna de Influenza Peru, 2016‐2018
Source: Influenza Other Respir Viruses. 2020 Apr 5;14(4):391–402. doi: 10.1111/irv.12737 (PMC7298283; doi:10.1111/irv.12737)
Supplement: Supplementary file 2 [file IRV-14-391-s001.docx]

Supplemental Figure A. Flow Diagram of Reported Acute Respiratory Illnesses during Active Surveillance among Fully Enrolled Participants, VIP Cohort, 2016-2018

[*Sent weekly during the flu season*]
In the past 7 days, have you felt sick with a cough, runny nose, body aches or feverishness?

[*If NO*] Thank you. Please remember to SMS "Sick" to this phone number if you start feeling ill.

[*If YES*]
How many days ago did you begin feeling sick? [*Records number*]
NEXT: We are going to ask you 7 questions.

Please answer yes or no if you've had any of the following 6 symptoms in the past 24 hours: Cough? Feeling feverish? Measured a temperature greater than 38 degrees Celsius? Sore throat? Runny nose? Body aches?

Finally, rate your ability to do activities today from 0 to 9, where 0 is unable to do any of your usual activities and 9 is able to do all of your usual activities. [*Records numbers*]

[*After 5 days*] Are you still feeling sick? [*If NO, repeat at 10 days; if still sick, repeat at 15 days*]

On the day you felt the sickest, rate your ability to do activities from 0 to 9 (repeat scale from above); During the illness did you miss any work hours because you were sick? [If yes, How many hours did you miss?] During the illness, did you switch shifts or reschedule your work hours because you were sick?
