# Supplementary material for: Prospective cohort study of influenza vaccine effectiveness among healthcare personnel in Lima, Peru: Estudio Vacuna de Influenza Peru, 2016‐2018
Source: Influenza Other Respir Viruses. 2020 Apr 5;14(4):391–402. doi: 10.1111/irv.12737 (PMC7298283; doi:10.1111/irv.12737)
Supplement: Supplementary file 3 [file IRV-14-391-s002.docx]

Supplemental Figure B. Southern Hemisphere, Trivalent Influenza Vaccine Formulations (2011-2018) and Hemmagglutination Inhibition Assay Targets, VIP Cohort, 2016-2018

							Study year vaccine southern hemisphere, trivalent influenza vaccine components		
		2011	2012	2013	2014	2015	2016	2017	2018
Year 1 (2016)	A(H1N1)pdm09	A/California/7/2009 (H1N1)pdm09-like virus	A/California/7/2009 (H1N1)pdm09-like virus	A/California/7/2009 (H1N1)pdm09-like virus	A/California/7/2009 (H1N1)pdm09-like virus	A/California/7/2009 (H1N1)pdm09-like virus	A/California/7/2009 (H1N1)pdm09-like virus	A/Michigan/45/2015 (H1N1)pdm09-like virus	A/Michigan/45/2015 (H1N1)pdm0º like virus
	A(H3N2)	A/Perth/16/2009 (H3N2)-like virus	A/Perth/16/2009 (H3N2)-like virus	A/Victoria/361/2011 (H3N2)-like virus	A/Texas/50/2012 (H3N2)-like virus	A/Switzerland/9715293/2013 (H3N2)-like virus	A/Hong Kong/4801/2014 (H3N2)-like virus	A/Hong Kong/4801/2014 (H3N2)-like virus	A/Singapore/INFIM' H-16-0019/2016 (H3N2)-like virus
	B(Victoria)/ B(Yamagata)	B/Brisbane/60/2008-like virus (B/Vic)	B/Brisbane/60/2008-like virus (B/Vic)	B/Wisconsin/1/2010 (B/Yam)	B/Massachusetts/2/2012-like virus (B/Yam)	B/Phuket/3073/2013-like virus (B/Yam)	B/Brisbane/60/2008-like virus (B/Vic)	B/Brisbane/60/2008-like virus (B/Vic)	B/Phuket/3073/2013-like virus (B/Yam)
		2011	2012	2013	2014	2015	2016	2017	2018
Year 2 (2017)	A(H1N1)pdm09	A/California/7/2009 (H1N1)pdm09-like virus	A/California/7/2009 (H1N1)pdm09-like virus	A/California/7/2009 (H1N1)pdm09-like virus	A/California/7/2009 (H1N1)pdm09-like virus	A/California/7/2009 (H1N1)pdm09-like virus	A/California/7/2009 (H1N1)pdm09-like virus	A/Michigan/45/2015 (H1N1)pdm09-like virus	A/Michigan/45/2015 (H1N1)pdm09-like virus
	A(H3N2)	A/Perth/16/2009 (H3N2)-like virus	A/Perth/16/2009 (H3N2)-like virus	A/Victoria/361/2011 (H3N2)-like virus	A/Texas/50/2012 (H3N2)-like virus	A/Switzerland/9715293/2013 (H3N2)-like virus	A/Hong Kong/4801/2014 (H3N2)-like virus	A/Hong Kong/4801/2014 (H3N2)-like virus	A/Singapore/INFIMH-16-0019/2016 (H3N2)-like virus
	B(Victoria)/ B(Yamagata)	B/Brisbane/60/2008-like virus (B/Vic)	B/Brisbane/60/2008-like virus (B/Vic)	B/Wisconsin/1/2010 (B/Yam)	B/Massachusetts/2/2012-like virus (B/Yam)	B/Phuket/3073/2013-like virus (B/Yam)	B/Brisbane/60/2008-like virus (B/Vic)	B/Brisbane/60/2008-like virus (B/Vic)	B/Phuket/3073/2013-like virus (B/Yam)
		2011	2012	2013	2014	2015	2016	2017	2018
Year 3 (2018)	A(H1N1)pdm09	A/California/7/2009 (H1N1)pdm09-like virus	A/California/7/2009 (H1N1)pdm09-like virus	A/California/7/2009 (H1N1)pdm09-like virus	A/California/7/2009 (H1N1)pdm09-like virus	A/California/7/2009 (H1N1)pdm09-like virus	A/California/7/2009 (H1N1)pdm09-like virus	A/Michigan/45/2015 (H1N1)pdm09-like virus	A/Michigan/45/2015 (H1N1)pdm09-like virus
	A(H3N2)	A/Perth/16/2009 (H3N2)-like virus	A/Perth/16/2009 (H3N2)-like virus	A/Victoria/361/2011 (H3N2)-like virus	A/Texas/50/2012 (H3N2)-like virus	A/Switzerland/9715293/2013 (H3N2)-like virus	A/Hong Kong/4801/2014 (H3N2)-like virus	A/Hong Kong/4801/2014 (H3N2)-like virus	A/Singapore/INFIMH-16-0019/2016 (H3N2)-like virus
	B(Victoria)/ B(Yamagata)	B/Brisbane/60/2008-like virus (B/Vic)	B/Brisbane/60/2008-like virus (B/Vic)	B/Wisconsin/1/2010 (B/Yam)	B/Massachusetts/2/2012-like virus (B/Yam)	B/Phuket/3073/2013-like virus (B/Yam)	B/Brisbane/60/2008-like virus (B/Vic)	B/Brisbane/60/2008-like virus (B/Vic)	B/Phuket/3073/2013-like virus (B/Yam)

Antigen targets for HI testing in each year
